# Circulating and tumor-infiltrating Tim-3 in patients with colorectal cancer

**DOI:** 10.18632/oncotarget.4112

**Published:** 2015-05-12

**Authors:** Benling Xu, Long Yuan, Quanli Gao, Peng Yuan, Peng Zhao, Huijuan Yuan, Huijie Fan, Tiepeng Li, Peng Qin, Lu Han, Weijia Fang, Zhenhe Suo

**Affiliations:** ^1^ Department of Oncology, The First Affiliated Hospital of Zhengzhou University, Zhengzhou, Henan, P. R. China; ^2^ Department of Cancer Biotherapy, The Affiliated Cancer Hospital of Zhengzhou University, Zhengzhou, Henan, P. R. China; ^3^ Department of Surgery, The Affiliated Cancer Hospital of Zhengzhou University, Zhengzhou, Henan, P. R. China; ^4^ Department of Oncology, The First Affiliated Hospital of Zhejiang University, Hangzhou, Zhejiang, P. R. China; ^5^ Department of Endocrinology, Henan Provincial People's Hospital, Zhengzhou, Henan, P. R. China; ^6^ Department of Pathology, Oslo University Hospital and Clinical Institute, Faculty of Medicine, University of Oslo, Oslo, Norway

**Keywords:** colorectal cancer, T cell exhaustion, PD-1, Tim-3

## Abstract

T-cell exhaustion represents a progressive loss of T-cell function. The inhibitory receptor PD-1 is known to negatively regulate CD8^+^ T cell responses directed against tumor antigen, but the blockades of PD-1 pathway didn't show the objective responses in patients with colorectal cancer (CRC). Thus, further exploring the molecular mechanism responsible for inducing T-cell dysfunction in CRC patients may reveal effective strategies for immune therapy. This study aims to characterize co-inhibitory receptors on T cells in CRC patients to identify novel targets for immunotherapy. In this study, peripheral blood samples from 20 healthy controls and 54 consented CRC patients, and tumor and matched paraneoplastic tissues from 7 patients with advanced CRC, subjected to multicolor flow cytometric analysis of the expression of PD-1 and Tim-3 receptors on CD8^+^ T cells. It was found that CRC patients presented with significantly higher levels of circulating Tim-3^+^PD-1^+^CD8^+^ T cells compared to the healthy controls (medians of 3.12% and 1.99%, respectively, *p* = 0.0403). A similar increase of Tim-3^+^PD-1^+^CD8^+^ T cells was also observed in the tumor tissues compared to paraneoplastic tussues. Tim-3^+^PD-1^+^CD8^+^ T cells in tumor tissues produced even less cytokine than that in paraneoplastic tissues. Functional ex vivo experiments showed that Tim-3^+^PD-1^+^CD8^+^ T cells produced significantly less IFN-γ than Tim-3^−^PD-1^−^CD8^+^ T cells, followed by Tim-3^+^PD-1^−^CD8^+^ T cells, and Tim-3^−^PD-1^+^CD8^+^ T cells, indicating a stronger inhibition of IFN-γ production of Tim-3^+^CD8^+^ T cells. It is also found in this study that Tim-3^+^PD-1^+^CD8^+^ T cell increase in circulation was correlated with clinical cancer stage but not histologic grade and serum concentrations of cancer biomarker CEA. Our results indicate that upregulation of the inhibitory receptor Tim-3 may restrict T cell responses in CRC patients, and therefore blockage of Tim-3 and thus restoring T cell responses may be a potential therapeutic approach for CRC patients.

## INTRODUCTION

CRC is the third most common cancer in women and the fourth most common in men worldwide [1]. Incidence of and mortality from CRC are increasing in China in recent years. Although the clinical management for CRC has become increasingly multi-modal, almost half of those undergoing curative resection die of metastatic disease and most CRC patients receive chemotherapy without clinical benefit [2]. It is very important to look for a new method to improve the effect of the treatment of CRC. Immune system, especially T lymphocytes, plays an important role in eliminating or controlling cancer [3], while the tumor microenvironment can be immuno-suppressive. Several lines of evidence support the finding that T cell antitumor function is impaired in CRC patients [4, 5]. Characterisation of the mechanism and molecules involved in the regulation of T cell responses will be valuable in the diagnosis and therapeutics of cancer.

T-cell exhaustion, a state of acquired T-cell dysfunction, is one of the different mechanisms proposed to explain such T cell impairment [6-11]. It was initially described in the context of chronic viral infection [12, 13], and recently reported in malignancies [8, 11]. The exhausted T cells show impaired cytotoxicity, reduced cytokine production and lose the capacity of proliferative.

Programmed death-1(PD-1) belongs to B7/CD28 family co-signalling molecules, a key immune-checkpoint receptor expressed by activated T cells mediating immunosuppression [14]. Inhibition of the expression of PD-1 on T cells may mediate antitumor activity. Topalian et al. [15] reported that no objective responses were observed in patients with CRC when anti-PD-1 antibody alone was applied as a therapeutic purpose. It is not clear right now that whether distinct T cell populations expressing different sets of inhibitory molecules exist. T cell immunoglobulin and mucin-domain-containing molecule 3(Tim-3) is a member of TIM family, and several lines of evidence support the role of Tim-3 as an inhibitory molecule that down-regulates effector Th1/Tc1 cell responses [16, 17]. It induces cell death in Th1 cells by binding to its ligand Galectin-9, and also induces peripheral tolerance.

To further determine whether other molecular pathways are involved in T cell dysfunction in CRC patients, we studied Tim-3 and PD-1 expression on CD8^+^ T cells of circulating, tumor-infiltrating lymphocytes (TILs) and paraneoplastic tissues from patients with CRC and investigated clinical relevance of co-inhibitory molecules in circulating and TILs in CRC.

## RESULTS

### Elevated Tim-3 expression on circulating CD8^+^ T lymphocytes in CRC patients

**Figure 1:**
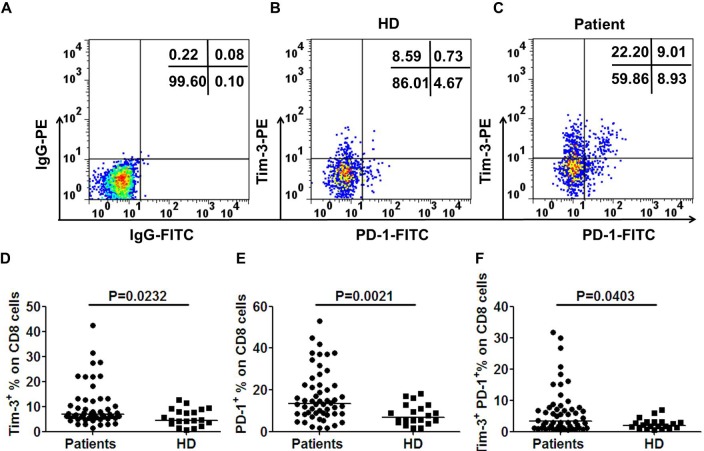
Tim-3 is up-regulated and coexpressed with PD-1 on CD8^+^ T cells in CRC patients To determine the percentage of Tim-3 and PD-1 in CRC patients, fresh whole blood was incubated with a combined anti-CD8, Tim-3 and PD-1 monoclonal antibodies. Acquired cells were first gated based on the expression of CD8. Within CD8^+^ population the fraction of cells expressing both Tim-3 and PD-1 was determined. Tim-3^+^ or PD-1^+^ percentage was calculated as percentage of CD8^+^ cells in whole blood samples. CD8^+^ T cells stained with PE-labeled and FITC-labeled IgG control antibodies were used to establish the threshold for identifying Tim-3^+^ or PD-1^+^ cells **A.**. Representative flow cytometric dotplots of a healthy volunteer **B.**, a CRC patient with stage III **C.**. Pooled data showing the percentage (%) of Tim-3^+^ **D.**, PD-1^+^ **E.** and Tim-3^+^PD-1^+^ **F.** expression on CD8^+^ T cells from CRC patients and healthy donors. Horizontal bars depict the median percentage of Tim-3 and/or PD-1 expression on CD8^+^ T cells. The P-values were calculated using the Wilcoxon signed rank test. *P* < 0.05 is considered statistically significant.

To better understand the exhausted T cells response to colorectal tumor mass, we first evaluated the expression of Tim-3 and PD-1 on circulating CD8^+^ T cells from CRC patients and healthy donors (Table 1 for demographics). Percentages of Tim-3, and PD-1 were calculated as a percentage of CD8 positive T cells. Fresh whole blood was incubated with a mixture of antibodies against CD8-PerCP-cy5.5, PD-1-FITC, and Tim-3-PE. A viability dye was used to exclude any dead cells from the analysis. Representative flow cytometric data of a normal healthy donor (HD, Figure 1B) and a CRC patient with stage III are shown in Figure 1C. The frequencies of Tim-3^+^ cells among CD8^+^ cells were significantly higher in CRC patients than those in normal volunteers (median, 6.78% *vs* 4.31%; *p* = 0.0232, Figure 1D). Consistent with previous study, a significantly higher level of circulating PD-1^+^ cells on CD8^+^ T cells in CRC patients were observed compared with normal volunteers (median, 13.45% *vs* 8.3%; *p* = 0.0021, Figure 1E).

**Table 1:** Patient characteristics

Variable	Category	Numbers
Total patients (n)		54
Median age in years (range)		61 (39-78)
Gender	male	40
	female	14
AJCC stage (2010)	I/II	23
	III/IV	31
Histologic differentiation	well/moderate	37
	poor	17
Weight loss	none	18
	≤10%	26
	>10%	10
Smoking status	current	32
	ex-smoker	8
	never	14

Next, we determined whether Tim-3 and PD-1 were expressed on identical or distinct T cell subsets. Analyzed by flow cytometry, the median percentage of Tim-3^+^PD-1^+^ cells among CD8^+^ T cells in CRC patients was significantly higher than that in the normal volunteers(3.12% *vs* 1.99%, *p* = 0.0403, Figure 1F).

### Higher expression of Tim-3 on TILs than that on paraneoplastic T lymphocytes within CRC patients

**Figure 2:**
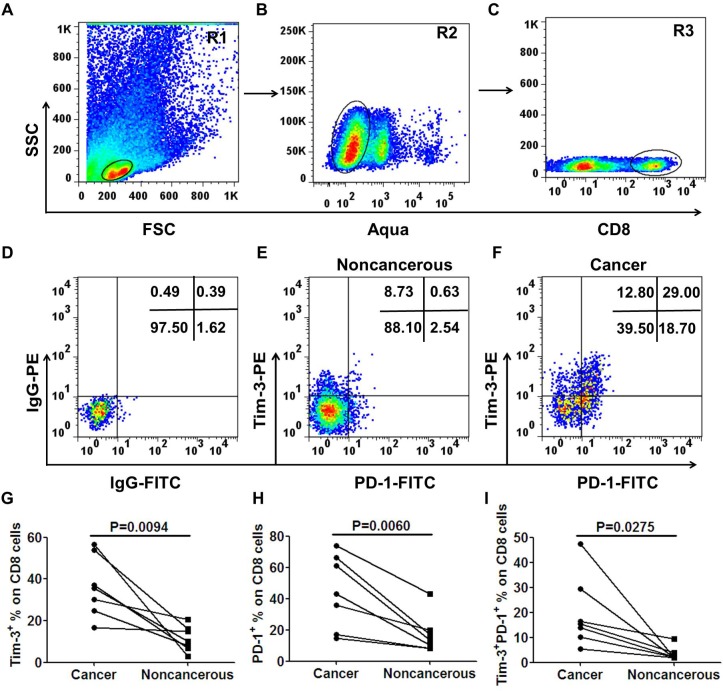
Significantly elevated levels of tumor-infiltrating Tim-3^+^PD-1^+^CD8^+^ T cells in advanced CRC patients Tumor tissues and matched surrounding non-cancerous tissues were obtained from 7 CRC patients with stage III. Tissues were disaggregated as specified in Materials and Methods and stained with fluorochrome-labeled antibodies against CD8, Tim-3 and PD-1. Acquired cells were first gated on R1 **A.**, within R1 the cells expressing Aqua^−^ were determined live cells (R2, **B.**), next the cells express CD8^+^ within R2 named R3 **C.**. Within R3 population the fraction of cells expressing both Tim-3 and PD-1 was determined. Representative scatter plots of Tim-3 and PD-1 expression on gated CD8^+^ T cells from a non-cancerous tissue **E.** and matched tumor tissue **F.**. Tim-3^+^ or PD-1^+^ percentage was calculated as percentage of CD8^+^ cells in tissues. Results showed a significantly higher percentage of Tim-3^+^ **G.**, PD-1^+^ **H.** and Tim-3^+^PD-1^+^ **I.** on tumor-infiltrating CD8^+^ T cells compared with matched non-cancerous tissues. The *P*-values were calculated using the paired *t*-test. *P* < 0.05 is considered statistically significant.

After we observed the increase in the level of circulating exhausted T cells in subjects with CRC, we determined to evaluate T cells from tumor tissues for a comprehensive immune characterization. To do this, we collected the fresh surgically excised tumors and paraneoplastic tissues of seven CRC patients with stage III and processed to single cell suspensions and stained with fluorochrome-conjugated antibodies against markers of exhausted T cells. The expression of Tim-3 and PD-1 on CD8^+^ T cell were then investigated. The representative flow cytometric dotplots were shown in Figure 2. As shown in the Figure 2G, compared with the matched paraneoplastic tissue, the percentage of Tim-3^+^ cells in tumor-infiltrating CD8^+^ T cells was significantly increased in the tumor tissues(36.37% *vs*. 11.37%, *p* = 0.0094). Like Tim-3, PD-1 was also expressed at elevated levels on tumor-infiltrating CD8^+^ T lymphocytes(44.56% *vs*. 17.13%, *p* = 0.0060, Figure 2H). Furthermore, Tim-3^+^PD-1^+^CD8^+^ T cells were significantly higher in tumor tissues than that in the paraneoplastic tissues(19.69% *vs*. 3.32%, *p* = 0.0275, Figure 2I ).

**Figure 3:**
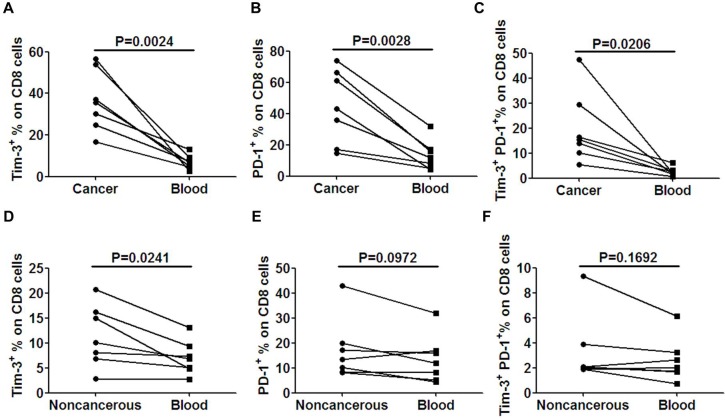
Tim-3 and PD-1 expression is higher on TILs than that on peripheral lymphocytes The expression levels of Tim-3 **A.**, PD-1 **B.** and Tim-3^+^PD-1^+^ **C.** were compared between cancer and peripheral blood. The expression levels of Tim-3 **D.**, PD-1 **E.** and Tim-3^+^PD-1^+^ **F.** were compared between non-cancerous tissues and peripheral blood. A paired *t*-test was used to compare the differences. *P* < 0.05 is considered statistically significant.

We then asked whether the expression of Tim-3 and PD-1 had any difference between TILs and circulating lymphocytes. To answer this question, Tim-3 and PD-1 expression on CD8^+^ T cells were analyzed. The percentages of Tim-3^+^ and PD-1^+^ cells on tumor-infiltrating CD8^+^ T cells were both significantly higher than their counterparts in blood(*p* = 0.0024, Figure 3A for Tim-3^+^, *p* = 0.0028, Figure 3B for PD-1^+^). Furthermore, Tim-3^+^PD-1^+^CD8^+^ T cells in TILs were also significantly higher than their counterparts in blood (*p* = 0.0206, Figure 3C). Next, the Tim-3 and PD-1 expressions on CD8^+^ T cells between noncancerous and blood were analyzed. The percentage of Tim-3^+^ cells on CD8^+^ T cells of noncancerous tissues was significantly higher than that in their counterparts in blood(*p* = 0.0241, Figure 3D), while there was no significant difference of PD-1^+^ (*p* = 0.0972, Figure 3E) or Tim-3^+^PD-1^+^ (*p* = 0.1692, Figure 3F) expression on CD8^+^ T cells between noncancerous and blood.

### Tim-3^+^PD-1^+^CD8^+^ T cells represent a dysfunctional T cell population

**Figure 4:**
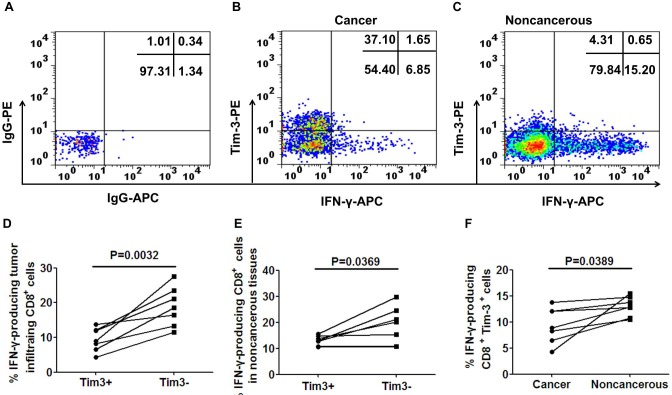
IFN-γ production of TILs and paraneoplastic CD8^+^ T cells classified by Tim-3 expression Representative scatter plots illustrating IFN-γ expression in cancer **B.** and noncancerous **C.** CD8^+^ T cells according to Tim-3 expression. Pooled data show the percent of IFN-γ producing cells in TILs **D.** and paraneoplastic CD8^+^ T cells **E.** according to Tim-3 expression from 7 patients. **F.** shows the percentage of IFN-γ producing Tim-3^+^CD8^+^ cells between cancer and noncancerous. A paired *t*-test was used to compare the differences. *P* < 0.05 is considered statistically significant.

We next compared the cytokine induction capability of Tim-3^+^ and Tim-3^−^ CD8^+^ T cells in tumor and paraneoplastic tissues ex vivo upon short stimulation (6h) with anti-CD3 (5ug/mL) [7, 18]. Percentages of IFN-γ-producing cells within Tim-3^+^ and Tim-3^−^ fractions were assessed after gating on live CD8^+^ T cells. As shown for one CRC patient in Figure 4A-4C and for seven CRC patients in Figure 4D-4F, Tim-3^+^CD8^+^ T cells produced significantly less IFN-γ than Tim-3^−^CD8^+^ T cells when TILs in tumors were examined (*p* = 0.0032, Figure 4D) and lymphocytes in noncancerous tissues were examined (*p* = 0.0369, Figure 4E). Furthermore, Tim-3^+^CD8^+^ T cells of the TILs produced significantly less IFN-γ than Tim-3^+^CD8^+^ T cells in the paraneoplastic tissues(*p* = 0.0389, Figure 4F).

**Figure 5:**
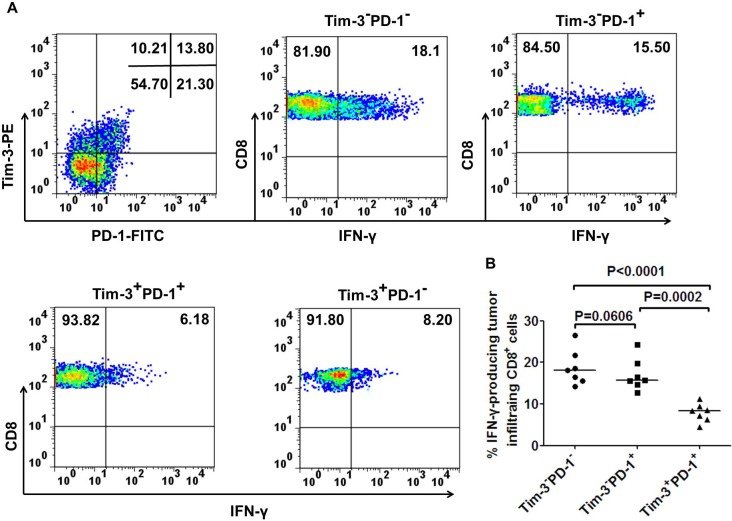
Co-expression of Tim-3 and PD-1 by tumor infiltrating CD8^+^ T cells defines a population of dysfunctional T cells Representative dot plots from one CRC patient **A.** and summary data for CRC patients (*n* = 7; **B.**) showing the percentage of IFN-γ-producing CD8^+^ T cells based on the expression of Tim-3 and PD-1 in tumor tissues after short ex vivo stimulation (6h) with anti-CD3. A paired *t*-test was used to compare the differences. *P* < 0.05 is considered statistically significant.

To better understand the major difference in cytokine production within TILs, we next investigated whether Tim-3^+^PD-1^+^ represented a more dysfunctional population than Tim-3^−^PD-1^+^, Tim-3^+^PD-1^−^ and Tim-3^−^PD-1^−^ cells within CD8^+^ TILs. As shown for one patient in Figure 5A and seven patients in Figure 5B, Tim-3^+^PD-1^+^CD8^+^ T cells produced significantly less IFN-γ than Tim-3^−^PD-1^−^CD8^+^ T cells(*p* < 0.0001). Tim-3^−^PD-1^+^CD8^+^ T cells produced a little lower IFN-γ than Tim-3^−^PD-1^−^CD8^+^ T cells, but it didn't reach significan difference(*p* = 0.0606). While, Tim-3^−^PD-1^+^CD8^+^ T cells produced higher IFN-γ than Tim-3^+^PD-1^+^CD8^+^ T cells(*p* = 0.0002). Notably, in two CRC patients we observed that Tim-3^+^PD-1^−^CD8^+^ T cells produced less IFN-γ than Tim-3^−^PD-1^−^CD8^+^ T cells, suggesting that Tim-3 expression alone defines a population of dysfunctional T cells, as previously reported for HIV-specific CD8^+^ T cells [17] and NY-ESO-1 specific CD8^+^ T cells [16]. In addition, we observed in these two patients that Tim-3^+^PD-1^+^CD8^+^ T cells produced significantly less IFN-γ than the Tim-3^+^PD-1^−^CD8^+^ T cells, suggesting that Tim-3^+^PD-1^+^CD8^+^ T cells represent a highly dysfunctional T cell population. Notably, the low frequencies of Tim-3^+^PD-1^−^CD8^+^ T cells did not allow us to evaluate their function in five out of the seven CRC patients tested. Collectively, our findings demonstrate that Tim-3^+^PD-1^+^CD8^+^ T cells are highly dysfunctional in terms of IFN-γ production in tumor tissues.

Because of the low frequencies of Tim-3^+^PD-1^+^CD8^+^ T cells, Tim-3^+^PD-1^−^CD8^+^ T cells and Tim-3^−^PD-1^+^CD8^+^ T cells in paraneoplastic tissues, we didn't analyze their IFN-γ induction. But we found that the Tim-3^−^PD-1^−^CD8^+^ T cells produced more IFN-γ in paraneoplastic tissues than that in TILs(*p* = 0.0075, Figure S1).

### Correlation of Tim-3^+^PD-1^+^CD8^+^ T cells with clinicopathological features

**Figure 6:**
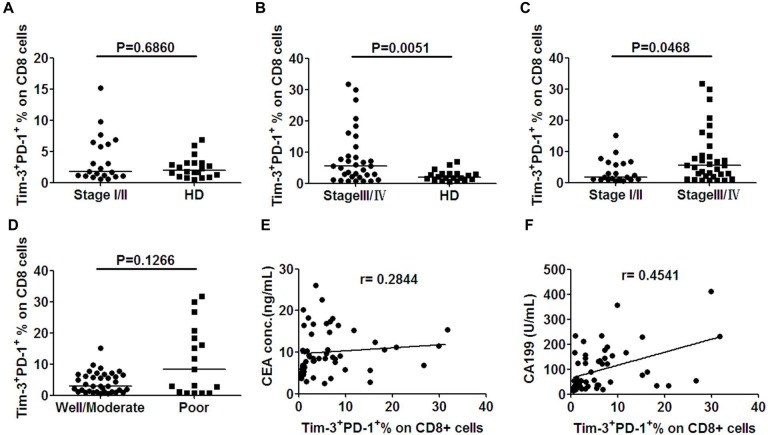
Elevated levels of circulating Tim-3^+^PD-1^+^CD8^+^ T cells in CRC patients correlated with clinical stage Whole blood drawn from patients prior to any therapy was analyzed for the presence of Tim-3^+^PD-1^+^CD8^+^ T cells. **A.** Results showed no significant difference of circulating Tim-3^+^PD-1^+^CD8^+^ T cells between cancer patients with stage I/II and healthy donors (HD). A significantly higher percentage of circulating Tim-3^+^PD-1^+^CD8^+^ T cells was observed in cancer patients with stage III/IV relative to HD **B.**, and stage III/IV relative to stage I/II **C.**. Scatter plots of Tim-3^+^PD-1^+^CD8^+^ T cells percentage in well/moderate *vs*. poor differentiation patients **D.**. Percentage of circulating Tim-3^+^PD-1^+^CD8^+^ T cells from all patients were analyzed to correlate with the serum concentration (before treatment) of cancer biomarker CEA **E.** and CA199 **F.** by unparametric spearman correlation analysis using GraphPad software. Bar denotes median in each group. *P* < 0.05 was considered as statistically significant.

Next, the association of circulating Tim-3^+^PD-1^+^CD8^+^ T cells with clinicopathological parameters was further analyzed in cancer patients. We divided patients by clinical cancer stage. The patients with stage I/II cancer contained the mildly increased percentage of Tim-3^+^PD-1^+^CD8^+^ T cells which did not reach the statistical significance when compared with HDs (median 1.74% *vs* 1.99%, *p* = 0.6860, Figure 6A). Significant difference was seen for the percentage of circulating Tim-3^+^PD-1^+^CD8^+^ T cells between healthy donors and patients with stages III/IV (median 1.99% *vs* 5.45%, *p* = 0.0051, Figure 6B). Significant difference was observed for the Tim-3^+^PD-1^+^CD8^+^ percentage in stage III/IV patients compared to patients with stage I/II (5.45% *vs* 1.74%, *p* = 0.0468, Figure 6C). No correlation was present between the histological grade and Tim-3^+^PD-1^+^CD8^+^ T cells (*p* = 0.1266, Figure 6D). In addition, we also analyzed the correlation between the Tim-3^+^PD-1^+^CD8^+^ T cells and serum concentration of cancer biomarker tested pre-treatment. No correlation was observed between the percentage of Tim-3^+^PD-1^+^CD8^+^ T cells and the serum concentration of cancer biomarker CEA (*r* = 0.2844, Figure 6E) and CA199 (*r* = 0.4541, Figure 6F).

## DISCUSSION

It has been reported that the immune status of patients with CRC patients was suppressed [19]. T lymphocytes form the major component of the adaptive immunity and tumor clearance. Although infiltrating CD4^+^ Th1 cells and CD8^+^ cytotoxic T cells sign a positive prognosis in CRC [20, 21], these lymphocytes often can't kill tumor cells. Recently research suggests that the expression of some cell-surface inhibitory receptors on T cells can induce T cell exhaustion.

PD-1 (CD279) is a key immune-checkpoint receptor expressed on activated T cells and appears to be associated with T cell exhaustion/dysfunction, but such dysfunction is not always reversable by blockade of PD-1 pathway, indicating that expression of other inhibitory receptors can also contribute to T cell exhaustion [22]. In this study, the expression of Tim-3 and PD-1 on circulating and TILs were studied. Tim-3 is a transmembrane protein constitutively expressed on Th1/Tc1 cells in mice and human [23]. Tim-3 expression correlates with a dysfunctional phenotype and blocking the interaction between Tim-3 and Tim-3's ligand rescues T cell proliferation and IFN-γ production in response to HCV-specific antigens [18]. In addition, Tim-3 maybe a stem cell marker in acute myeloid leukemia(AML) [24].

In the present study, we first observed that Tim-3 and PD-1 expression was up-regulated on circulating CD8^+^ T cells in CRC patients, a finding consistent with previously study that the expression of PD-1 is significantly higher in patients with cancer relative to the levels in healthy controls, such as T-follicular helper cells lymphoma [25], head and neck cancer [26], oral squamous cell carcinoma [27], and ovarian cancer [28]. Next analysis showed that Tim-3^+^PD-1^+^CD8^+^ T cells in CRC patients are significantly higher than that in healthy donors. Furthermore, in contrast to the low level expression of Tim-3^+^PD-1^+^CD8^+^ on peripheral blood lymphocytes, Tim-3^+^PD-1^+^CD8^+^ T cells represent the larger population of TILs, and this cell population in TILs is also higher than that in the paraneoplastic tissues. These cells are more dysfunctional than Tim-3^−^PD-1^−^CD8^+^ T cells *ex vivo*. We found no significant difference in terms of IFN-γ production between the Tim-3^−^PD-1^−^CD8^+^ and Tim-3^−^PD-1^+^CD8^+^ T cells, suggesting that PD-1 up-regulation alone without Tim-3 up-regulation is not directly associated with IFN-γ secretion. This observation is in line with the previous study that PD-1 acts as a regulator of CD8^+^ T cell expansion upon chronic antigen exposure and has no major impact on cytokine secretion [29]. In two CRC patients with higher levels of Tim-3^+^CD8^+^ T cells, we found that Tim-3^+^PD-1^−^CD8^+^ T cells produced less IFN-γ than the Tim-3^−^PD-1^−^CD8^+^ T cells, suggesting that Tim-3 up-regulation alone by tumor antigen-specific CD8^+^ T cells defines a group of dysfunctional T cells independently of PD-1 up-regulation. Importantly, the Tim-3^+^PD-1^+^CD8^+^ T cells produced significantly less IFN-γ than the Tim-3^+^PD-1^−^CD8^+^ T cells, supporting the notion that Tim-3^+^PD-1^+^CD8^+^ T cells represent a more dysfunctional population than the Tim-3^+^PD-1^−^CD8^+^T cells. The low frequencies of Tim-3^+^PD-1^−^CD8^+^ T cells did not allow us to extend this observation to the other CRC patients included in our study. Interestingly, one study in melanoma patients also shown that co-expression of inhibitory receptors PD-1 and Tim-3 by NY-ESO-1 specific CD8^+^ T cells was associated with lower T cell functions [16]. Our findings are consistent with a previous study that Tim-3^+^PD-1^+^CD8^+^ T cells are more dysfunctional in TILs and Tim-3^−^PD-1^−^CD8^+^ T cells are major producers of IFN-γ in the TILs of MC38 and CT26 tumor models( colon adenocarcinomas) in mice [30]. Our findings further add to this observation and support that co-expression of Tim-3 and PD-1 is a marker of tumor-induced T cell dysfunction in patients with advanced CRC.

Our results suggest that even though TILs may accumulate within tumor tissues in CRC patients, their efficacy may be limited by inhibitory factors within tumors. Given that Tim-3 negatively regulates the IFN-γ-mediated Th1 responses, the use of anti-Tim-3 monoclonal antibody may complement the therapies relieving T cell anergy/exhaustion/tolerance. Ngiow SF, et al. [30] reported that anti-Tim-3 alone reduced the frequency of Tim-3^+^ T cells in the TILs of CT26 and MC38 tumor models, and suggested that combination of anti-Tim-3 with anti-CTLA-4 and anti-PD-1 might be well tolerated and very effective. So, the relative kinetics of TILs accumulation versus their rate of exhaustion within tumors will ultimately determine the overall antitumor efficacy.

Although our studies have shown that Tim-3^−^PD-1^−^CD8^+^ T cells produced more IFN-γ than Tim-3^+^PD-1^+^CD8^+^ T cells within TILs, the IFN-γ induction level is still lower than that produced by Tim-3^−^PD-1^−^CD8^+^ T cells in paraneoplastic tissues, supporting the hypothesis that the tumor microenvironment may up-regulation of inhibitory receptors. Thus exploring factors upregulating such inhibitory receptors within the tumor microenviroment will be worthy of further mechanism study of T cell exaustion.

In addition, Tim-3 expression correlates not only with a dysfunctional but also with senescent phenotype [18]. Accumulating evidence indicates that cancer is an age-related disease [31, 32]. The incidence of common cancers including breast, lung, colorectal, and ovarian carcinomas as well as of some types of leukemia is increased exponentially with age [33, 34]. The immune system can recognize and eliminate not only cells that are prone to undergo malignant transformation, but also senescent cells, thus playing an imptortant role in the control of organismal aging. In addition, it has recently become clear that some immunosuppression (such as rapamycin and other rapalogs), which for a long time have been viewed as (and used in the clinic) pure immunosuppressants, can mediate robust immunostimulatory functions, at least in some circumstances [32, 35-39], indicating a complex of immune regulation in tumors. Therefore, further studies are required to address these questions.

In our present study it is shown that higher Tim-3^+^PD-1^−^CD8^+^ T cells are found in TILs of some patients, while others are not. It is not clear whether the expression of Tim-3 is associated with some gene mutation or pathological type. However, a recent research reports that the increased Tim-3 expression in AML may be linked to the mutations in CEBPA [24], and this finding may encourage us to study such a possibility in CRC in the next step of our study.

One novel finding is that the percentage of Tim-3^+^PD-1^+^CD8^+^ T cells was correlated with clinical stage. The significant difference was observed between patients with advanced tumor and healthy donors while patients with stage I/II had a moderately increased percentage of circulating Tim-3^+^PD-1^+^CD8^+^ T cells, although we did not observe significant correlation between the Tim-3^+^PD-1^+^CD8^+^ T cells and histologic grade and serum concentrations of cancer biomarker CEA and CA199. The correlation between Tim-3^+^PD-1^+^CD8^+^ T cells with clinical stage indicates Tim-3^+^PD-1^+^CD8^+^ T cells may stimulate tumor progression. A recent study shows the increased percentage of Tim-3 and PD-1 on circulating CD4^+^ and CD8^+^ T cells is associated with dysfunction of cell-mediated immunity after colorectal cancer operation, while the relationship between the expression of Tim-3^+^PD-1^+^CD8^+^ T cells with clinicopathological parameters, the expression and function of Tim-3 and PD-1 in TILs have not been determined in their study [40]. In addition, our results are consistent with the change of Tim-3^+^PD-1^+^CD8^+^ T cells seen in mouse CRC models showing the appearance of Tim-3^+^PD-1^+^CD8^+^ T cells dominated the majority of CD8^+^ TILs only at a later tumor stage [30] and PD-1 and Tim-3 co-expression increased during AML progression [10]. More studies are apparently needed to elucidate the relevance between Tim-3^+^PD-1^+^CD8^+^ T cells with prognosis and overall survival in CRC, which may lay a theoretical foundation for Tim-3-targeting therapies in this type of cancer.

In summary, our data demonstrate that Tim-3^+^PD-1^+^CD8^+^ T cells represent a highly dysfunctional population of tumor-induced T cells in patients with advanced CRC and this cell population is significantly correlated with higher clinical stage, indiating strongly for further studies on the molecular mechanism of Tim-3-mediated immunosuppression, which may lead to novel treatments such as combining anti-Tim-3 with anti-PD-1 in CRC patients.

## MATERIALS AND METHODS

### Ethics statement

This study was approved by The Institute Ethical Committee of The Affiliated Cancer Hospital of Zhengzhou University, China. Written informed consents were obtained from all patients.

### Patients

Patients with CRC diagnosed on the basis of pre-operative staging and laparotomy findings, were approached for enrollment between 2012 and 2013 at the Department of Surgery, The Affiliated Cancer Hospital of Zhengzhou University, China. Fifty-four patients diagnosed with primary CRC, and had not been previously treated were qualified and enrolled in this study. Peripheral blood samples were collected from each patient before treatment. The median age of patients in this study was 61 years (range 39-78 years). Samples were also obtained from twenty healthy gender- and age-matched controls (median age 58 years). The clinical stage was classified according to the American Joint Committee on Cancer (AJCC) Staging Manual, Seventh Edition (2010). The following clinicopathological characteristics were recorded: age, sex, weight loss, histological grade, and smoking status at the time of blood sample collection (Table 1).

### Phenotypic analysis

Fresh venous blood was collected from patients or healthy donors with EDTA-coated vacutainer tubes. Specific antibodies against PD-1-FITC (Biolegend), CD8-PerCP-cy5.5 (BD Pharmingen™), Tim-3-PE (BD Pharmingen™) were used. A violet amine reactive dye (Invitrogen) was used to assess the viability of the cells. Fresh venous blood samples labeled with corresponding fluorochrome conjugated non-immune isotypes were taken as negative controls. Briefly, 50 μL of blood was mixed with 10μL of each antibody and was incubated on ice for 20 min in dark. After incubation, each sample was mixed with 2 mL of 1X BD FACS^TM^ lysing solution (BD Biosciences) and incubated at room temperature for 15 min. Samples were washed with FACS buffer (5% BSA in PBS, 0.09% sodium azide). The pellets were resuspended in 300 μL of FACS buffer and acquired on a BD FACS Aria II flow cytometer, and the data were analyzed with FlowJo software (TreeStar Inc).

### Isolation of TILs

To characterize the leukocytes present in the tumor site, the fresh human tumor samples and matched paraneoplastic tissues from CRC patients with stage III were cut into small pieces(3∼5mm^3^), and digested with collagenase I (1ug/mL, Sigma-Aldrich, St.Louis, MO, USA), collagenase IV (1ug/mL, Sigma-Aldrich, St.Louis, MO, USA) and DNase (25ug/mL; Sigma-Aldrich) at 37°C for 1∼1.5 h. The tissue homogenates were filtered using a 70-um cell strainer (Falcon; BD Biosciences) and subjected to the density centrifugation. The leukocyte's viability was evaluated by Trypan blue exclusion.

### Intracellular IFN-γ staining assay

To evaluate the generation of IFN-γ producing cells in T cells, isolated TILs and T cells in noncancerous tissues were stimulated with anti-CD3 (5 ug/ml, eBioscience, San Diego, CA, USA) for 6 h and subsequently analyzed for IFN-γ[7, 18]. After 2h of incubation, Brefeldin A (Sigma-Aldrich) was added to the culture medium (10 ug/mL). Cells were then stained with CD8-PerCp-Cy5.5, Tim-3-PE, PD-1-FITC, and intracellularly stained with IFN-γ-APC (Miltenyi Biotec) antibodies. Five hundred thousand events were collected during flow cytometric analysis.

### Statistical analysis

Statistical analysis was performed on GraphPad Prism 5.0 software (GraphPad Software, United States). Mann-Whitney test and unparametric Spearman test were used to assess the differences and correlation between the study groups respectively. A paired t-test was used to compare the Tim-3 and PD-1 expression in tissues, as well as to evaluate differences in IFN-γ production on T cells subsets in cancer and noncancerous tissues. *P* value < 0.05 was considered statistically significant.

## SUPPLEMENTARY MATERIAL FIGURE


