# Supplementary material for: Circulating and tumor-infiltrating Tim-3 in patients with colorectal cancer
Source: Oncotarget. 2015 May 12;6(24):20592–603. doi: 10.18632/oncotarget.4112 (PMC4653028; doi:10.18632/oncotarget.4112)
Supplement: Supplementary file 1 [file oncotarget-06-20592-s001.pdf]

Circulating and tumor-infiltrating Tim-3 in patients with colorectal cancer

Supplementary Material

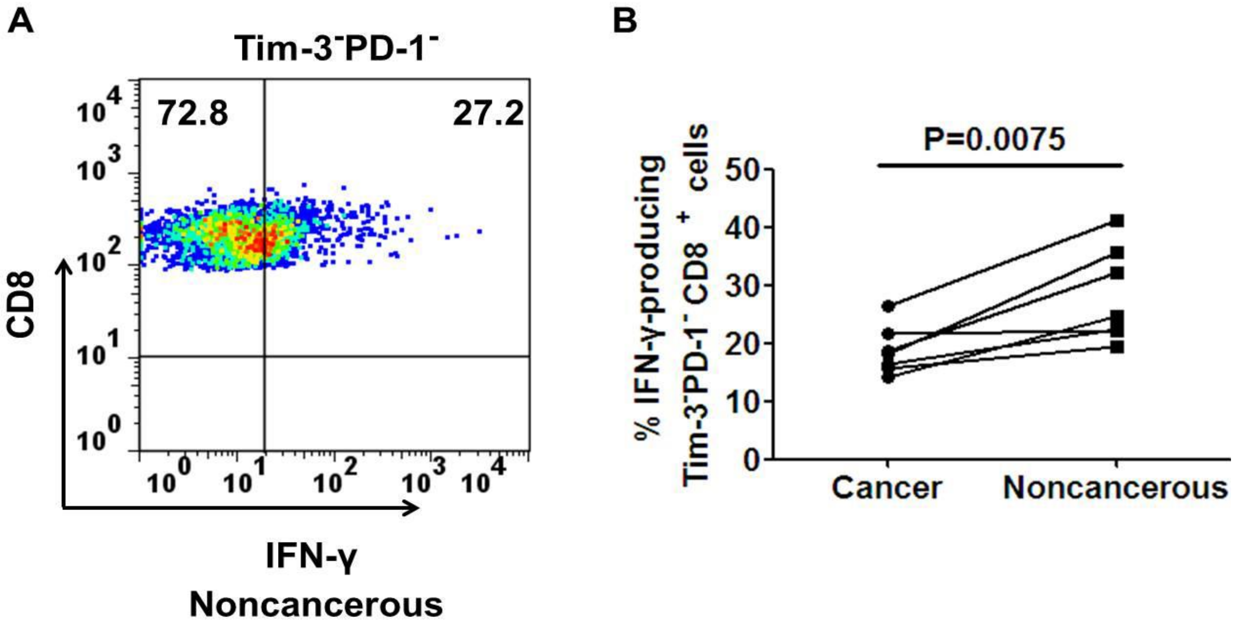

Supplemental Fig 1: The levels of IFN-γ-producing Tim-3$^-$PD-1$^-$CD8$^+$ T cells in tissues. Representative dot plots of the percentage of IFN-γ-producing Tim-3$^-$PD-1$^-$CD8$^+$ in noncancerous tissues (A). Pooled data showing the percentage of IFN-γ-producing Tim-3$^-$PD-1$^-$ CD8$^+$ T cells from tumor tissues and paraneoplastic tissues (B). The P-values were calculated using the paired *t*-test. P<0.05 is considered statistically significant.
